# Combined ANN/EVOP Factorial Design Approach for Media Screening for Cost-effective Production of Alkaline Proteases from *Rhizopus oryzae* (*SN5*)/NCIM-1447 under SSF

**DOI:** 10.1186/s13568-020-00996-7

**Published:** 2020-03-27

**Authors:** Sangeeta Negi, Sapna Jain, Anand Raj

**Affiliations:** Department of Biotechnology, Motilal Nehru National Institute of Technology Allahabad, Prayagraj, UP 211004 India

**Keywords:** *Rhizopus oryzae*, EVOP, Artificial neural networks (ANN), Alkaline protease, Wheat bran

## Abstract

In order to achieve high yield of fungal protease in a very cost effective way and to meet its increased market demand, current study deals with the screening of various agro-wastes as carbon source for the production of protease from *Rhizopus oryzae* (*SN5*)/NCIM-1447 under solid state fermentation. Substrates and culture parameters such as wheat bran, soybean meal, black-gram husk, rice husk, mixture of wheat bran, soybean meal, nitrogen sources, pH, temperature and incubation time were first optimized with one factor at time strategy and then EVOP factorial and yield of alkaline protease was achieved 412.8 U/gds at 28 °C and pH = 6 after 72 h of fermentation taking wheat bran and soybean as a substrate in 4:1 ratio. Further artificial neural networks (ANN), was trained with data of EVOP and yield of protease was enhanced up to 422.6 U/gds with wheat bran: soyabean in ratio of 70:30, pH 6.2 at 30 °C. The evolved process and *Rhizopus oryzae* (*SN5*)/NCIM-1447 strain would be promising for protease production at industrial scale at low cost.

## Introduction

Proteases or peptidyl-peptide hydrolases (EC: 3.4.21-24 and 99) catalyze the total hydrolysis of proteins by acting on their peptide bonds. They are able to catalyse both hydrolytic and synthetic reactions, based on substrate, pH and other physicochemical conditions. Alkaline proteases occupy a pivotal position with respect to their applications in both physiological and commercial fields. They are mainly used in food industry as probiotics, in food processing, in beverages etc., as antimicrobial agent in medicine, textile manufacturing, detergent, food-processing, paper and pulp industries, leather industry, etc.

Proteases posses all functions, such as high microbial activity, can make protecting layer over intestinal wall against pathogens and can also modulate host immune system, which makes them best food supplements for all purposes. Diversified proteases are expressed by many microorganisms, may be very specific or nonspecific that hydrolyse variety of proteins associated with innate immunity to proteases that are extremely precise and specific in their mode of action (Craik et al. 2011; Potempa and Pike. 2009). They are the part of various important biological functions i.e., cardiovascular, nervous, gastrointestinal and immune systems (Mazaa et al. 2012; Donnelly et al. 2011) etc., therefore used as medicine through functional food also.

The future of protease enzymes in the pharmaceutical industry shows promising growth in forthcoming years. The global protease enzymes market is envisaged to grow at a healthy CAGR of 5.3%. From 2014 to 2019, protease consumption in China is expected to elevate at a CAGR of 6%, reaching up to USD 272.6 million by 2019 (Global Trends & Forecasts to 2019). In order to satiate the increasing demand, it is extremely necessary to formulate cost-effective methods for production of highly active and stable proteases.

Proteases sources are animals, plants and microorganisms. But on the basis of various economical, technological and ethical issues, microorganisms are considered as the best sources of proteases (Sharma et al. 2019). Many past works have been done for the production of proteases by strains of *Bacillus* (Sattar et al. 2011), *Rhizopus* (Benabda et al. 2019; Pandey et al. 2016) and *Penicillium* (Djamel et al. 2009; Omrane et al. 2018). Fungi elaborate a wider variety of enzymes than bacteria. The fungal proteases are active over a wide pH range (pH 4.0–11.0) and exhibit broad substrate specificity. They are also used in the pharmaceuticals, medical diagnosis and in the detergent industry as additives (Mamo and Assefa 2018). Fungal alkaline proteases are also used in food protein modification and processing (Neethu et al. 2016).

In present investigation a locally isolated strain *Rhizopus oryzae* (SN5) NCIM has been used for the production of proteases. In order to reduce the production cost of enzyme, various agro waste and low cost, readily available biomass has been used under solid state fermentation (SSF). Other important factors which impart great impact on the yield of enzyme are culture parameters such as temperature, pH, inducers, carbon and nitrogen sources and their respective concentration. Therefore through various mathematical and computational optimization techniques, optimal combination of various culture parameters and substrate ratio have been achieved by researchers, such as one-variable-at-a-time, RSM (Milala et al. 2016), EVOP-factorial design technique (Negi and Banerjee 2006; Pandey et al. 2016), Artificial neural network (ANN) (Román et al. 2011; Vats and Negi 2013), genetic algorithm etc. ANN has been preferred because of their proven advantages over other mathematical and computational method in order to get optimum physicochemical conditions for optimum yield of fermentation products (Yadav et al. 2013; Vats and Negi 2013). An artificial neuron network (ANN) is a computational model based on the structure and functions of biological neural networks and can be used to approximate any function that can depend on a large number of inputs. Neural networks are massively parallel and distributed processing systems representing a new computational technology built on the analogy to the human information processing system, i.e. neural networks. Basic parts of ANN are neurons, which receives input from some other nodes or from an external source and computes an output. Each input has an associated weight (w), which is assigned on the basis of its relative importance to other inputs, all neuron in network has a bias(b) associated with it. Three-layer Multilayer Feedforward Neural Network (MFNN) structure in which the *input layer* and the *output layer* are directly interconnected with the intermediate single *hidden layer* have the inherent capability to carry out any arbitrary input–output mapping with very high efficiency. When trained on examples of observation data, the networks can learn the characteristic features “hidden” in the examples of the collected data and even generalize the knowledge learnt. In present investigation ANN has been used for the optimization of the important physicochemical parameters such as combinational ration of different substrate as carbon source, pH and incubation temperature to achieve optimum yield of protease enzyme from locally isolated *Rhizopus oryzae SN5*.

## Materials and methods

### Chemicals

The chemicals used in this study were all of analytical grade and all the substrates viz. wheat bran, rice husk, soybean meal and black gram husk were purchased from local market at Teliarganj, Allahabad-211004, India.

### Maintenance of culture and inoculum preparation

For the present work, *Rhizopus oryzae (SN5)/*NCIM-1447strain isolated from compost soil obtained from Ranikhet, Uttrakhand, India was utilized. This strain was characterised from NCIM, Pune India and deposited. Strain Seq 286_NC111119 showed closest homology with Rhizopus sp. (closer to oryzae). This strain was maintained on Potato Dextrose Agar (PDA) slants at 4 °C. For preparation of inoculum, 25 mL of sterile distilled water was added to 5 days old slant and aseptically scraped with an inoculating loop. This suspension of spores was used as inoculum for the preparation of induced culture.

### Preparation of induced culture

Medium for preparation of induced culture contained 1% casein, 0.25% glucose in modified Czapek–Dox broth. This medium was autoclaved and then inoculated with 2% of the spore suspension containing approximately 3.2 × 10^6^ spores/ml. The medium was incubated at 28 °C for 6 days. After the complete growth of the organism, the flask was placed on a shaker to make a homogenous suspension of spores.

### Fermentation set up

The fermentation was carried out in 500 mL Erlenmeyer flasks, each carrying 5 g of different substrates viz. wheat bran, soybean meal, a mixture of wheat bran and soybean meal in ratio 4:1, black-gram husk and rice husk, respectively. The substrates were soaked in 10 mL of Czapek–Dox salt solution with pH 6 and autoclaved. This preparation was then inoculated with 1 mL of the induced spore suspension containing about 4.3 × 10^7^ spores and kept in incubator at 28 °C. Fermentation was carried out in different experimental conditions changing one parameter at a time.

### Extraction of crude enzyme

The fermented substrate was soaked in 50 mL of 0.1 M phosphate buffer and kept on a shaker at 120 rpm for 2 h. The substrate was filtered through muslin cloth and the extract was centrifuged at 10,000 rpm at 4 °C for 10 min for removal of spores and other insoluble debris. The supernatant or crude enzyme extract was stored at 4 °C for measurement of protease activity.

### Protease assay

Protease activity was measured through modified caseinolytic method (Walter 1984). 0.65 mL of glycine–NaOH buffer with pH 8 was incubated with 0.05 mL of enzyme solution for 10 min. 2 mL of 2% casein solution was then added and the reaction mixture was incubated at 37 °C for 20 min. The reaction was ceased by adding 0.1 mL of 1 M HCl and the non-hydrolysed casein was precipitated by 5 mL of 5% Trichloroacetic acid. The precipitated casein was removed through centrifugation at 10,000 rpm for 5 min. The protein concentration was measured in 0.5 mL of the supernatant through Lowry method using BSA as standard (Lowry et al. 1951). One unit of enzyme activity is defined as the amount of enzyme that liberates peptide fragments equivalent to 1 mg of BSA per minute under the assay conditions (Patil and Shastri, 1981).

### Effect of process parameters on enzyme production: carbon sources, incubation time, pH, temperature

Various process parameters such as temperature, carbon sources, incubation time, pH, carbon and nitrogen sources, were studied to monitor their effect on alkaline protease production in solid-state fermentation through one at a time first and then with EVOP factorial design technique.

To screen carbon source, 5 g each of different agro-industrial wastes such as wheat bran, rice husk, black-gram husk, soybean meal and a mixture of wheat bran and soybean meal in ratio 4:1 (in g) were used as substrate in different combinations for fermentation. The fermentation flasks were incubated for 24, 48, 72, 96, 120 and 144 h and protease activity was measured according to the procedure described earlier. The ratio of Wheat Bran and Soybean Meal was changed as follows- 4.5:0.5, 4:1, 3:2, 2:3 (in g) and fermentation was done as described before. In order to optimum nitrogen supplement to achieve max yield Czapek–Dox salt solution was supplemented with sodium nitrate (1%), ammonium nitrate (1%), peptone (1%), yeast extract (1%) and potassium nitrate (1%) to study the impact of different nitrogen sources on alkaline protease production.

Physical parameters like initial pH and temperature were optimized by maintaining the pH of Czapek–Dox broth using 1 N HCl/1 N NaOH as 4, 5, 6, 7 and 8 before autoclaving media and temperature was kept 28, 30, 35 and 37 °C by keeping the fermentation flasks in different incubators pre-set at respective temperature.

### Process parameters optimization through EVOP factorial design technique

The EVOP methodology was applied to determine the optimum levels of three parameters (ratio of wheat bran to soybean meal, pH and temperature) in different experiments. Firstly, the control experimental conditions (A1 and A6) were selected based on the results obtained from one factor at a time optimization. Then, new experimental conditions with higher and lower search levels of parameters were selected. The experimental design for three variables system is shown in Table 1. Thirdly, fermentation was carried out as described earlier at higher and lower search levels and all the experiments were repeated for two cycles. The yield of alkaline protease in cycle I and cycle II was measured following the procedure mentioned earlier. The differences in protease yield in cycle I and cycle II were calculated along with the averages in order to estimate the effects and error limits. The magnitude of the effects, error limits and changes in the main effect were inspected as per the decision making procedure to arrive at the optimum.

**Table 1:** Experimental design for three variables system

Parameters	A1	A2	A3	A4	A5	A6	A7	A8	A9	A10
Temperature/°C	0	–	–	+	+	0	+	–	+	–
pH	0	–	+	–	+	0	+	–	–	+
WB +SM ratio	0	–	+	+	–	0	+	+	–	–
Response	a1	a2	a3	a4	a5	a6	a7	a8	a9	a10

### Decision making

Once all the calculations for effects and error limits are completed, it is extremely necessary to determine whether any changes in the control level will help to enhance the objective function and if the changes are required then the next step is to identify the desired direction of change. For this study, magnitudes of the effects were compared to those of the error limits. If all or any of the effects are higher than the error limits, alterations in the experimental conditions may yield better results.

### Optimization of process parameters through ANN method

Data selected for optimization.

Data used in ANN was output of Evop-Factorial design technique and one factor at a time i.e., pH, incubation temperature and different percentage of two different substrate combination of Wheat bran (WB)and Soybean mil (SM) (Table 2).

**Table 2:** shows the data selected to optimize the protease activity

Temperature	WB % in (WB + SM)	pH	Protease activity
28	80	6	398.03
26	70	4	144.45
26	90	8	49.38
30	90	4	93.74
30	70	8	144.1
28	80	6	398.47
30	90	8	195.13
26	90	4	114.85
30	70	4	106.97

The data selected for optimization was used to fit a nonlinear regression:

$$\begin{aligned} {\text{Proteaseactivity}} = & - \;2299.6275 + 7.896875*{\text{x}}(1) - 0.591625*{\text{x}}(2) \\ & + 839.270625*{\text{x}}(3) - 69.7646875*{\text{x}}(3)^{2} \\ \end{aligned}$$

where: x(1) = temperature value, x(2) = WB percentage in the WB and SM substrate mixture, x(3) = pH value.

#### Optimization methodology

This analysis was carried out in MATLAB, Windows 8 operating system, Intel R CPU 2.53 GHz, 8.00 GB of RAM. The input data and target data was the data collected from the Table 2, which was fed into the designed neural network for training. The speed of this process varies according to the specifications of the system. There are different types of neural network available for training and optimization. However, we had chosen to solve this particular problem using feed forward network and trained by back propagation algorithm. The network flow is only in one direction. There was no feedback because we wanted to create a simple network for this problem. This network used supervised learning so input and output data were fed to the network. In case of unsupervised learning, only input has given and linear equations were built such that maximum correlation coefficient was achieved. Signals flow forward and errors are propagated backward to ensure that errors were reduced and R square value increased closer to 1. Random weights were initialized and changed in each run of each iteration to made change in learning process. The objective was to minimize the errors. The different parameters considered in the neural network included training functions, performance measures, number of layers, number of hidden layers and neurons, and transfer function.

#### Network architecture and training of MFNN

**Fig. 1:**
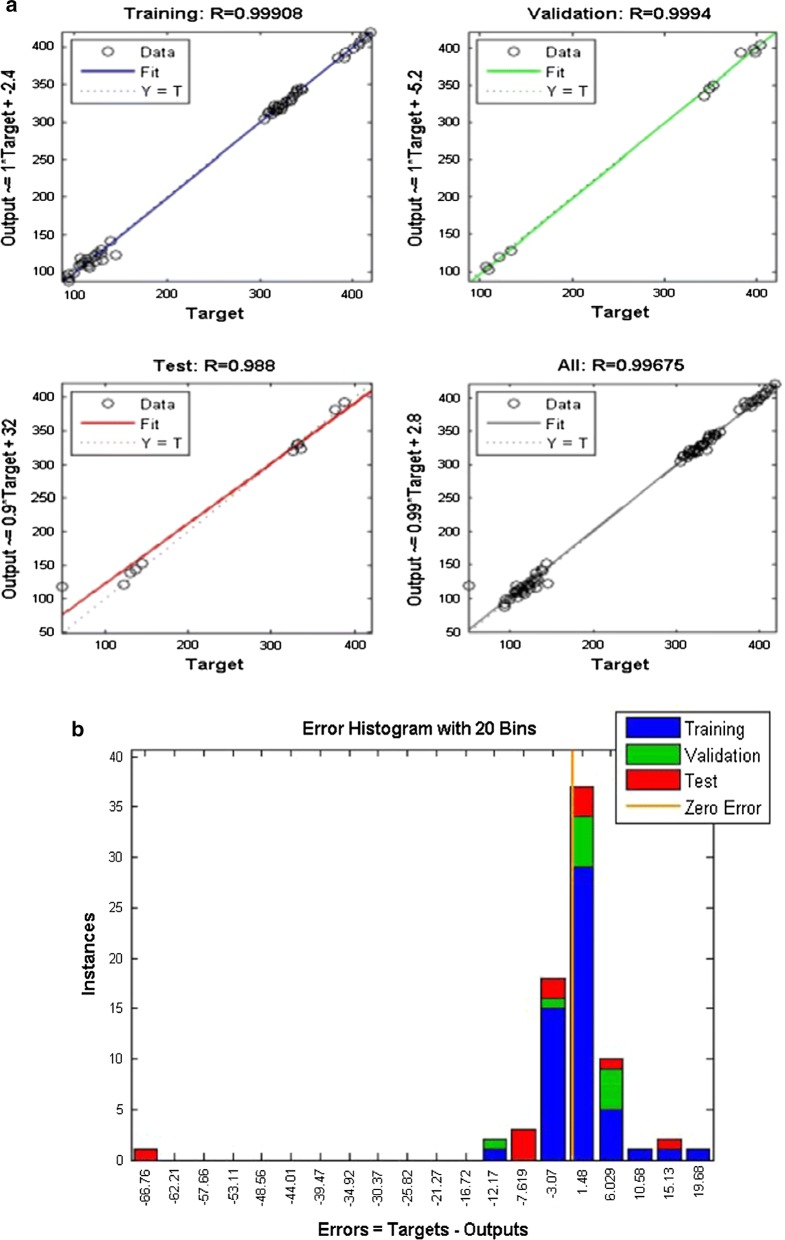
**a** The graphical depiction of the regression results of training, validation and testing. **b** Error histogram plot of training of Network

A three layer (3-10-1) MFNN was designed using MATLAB: 1 input layer (3 neurons), 1 hidden layer(10),1 output layer(1 neuron). Layers were kept fully connected means each neuron in next layer has been connected to each neuron in previous layer. The sigmoidal function was used as transfer function of neurons in hidden layer and linear function was used as transfer function of output layer. Sigmoidal function gives more accuracy with backpropagation algorithm. ‘Trainlm’ was used for training the MFNN because it is a fast and stable algorithm (M63). The network was trained for 64 times to find best fit to the data. The training results of The R square value was found 0.9967 for overall, 0.99909 for Training, 0.9994 for validation and 0.988 for text, which is very close to one and acceptable for good fitting. Performance of MFNN is shown in Fig. 1a, b.

#### Calculating network output

The trained neural network was used to generate protease activity at various input values of temperature, WB%, and pH value setting constraints.

1. 26 ≤ temperature ≤ 30 at increment of 1 °C.
2. 70 ≤ WB% ≤ 90 at increment of 5.
3. 4 ≤ pH ≤ 8 at increment of 0.2.

**Fig. 2:**
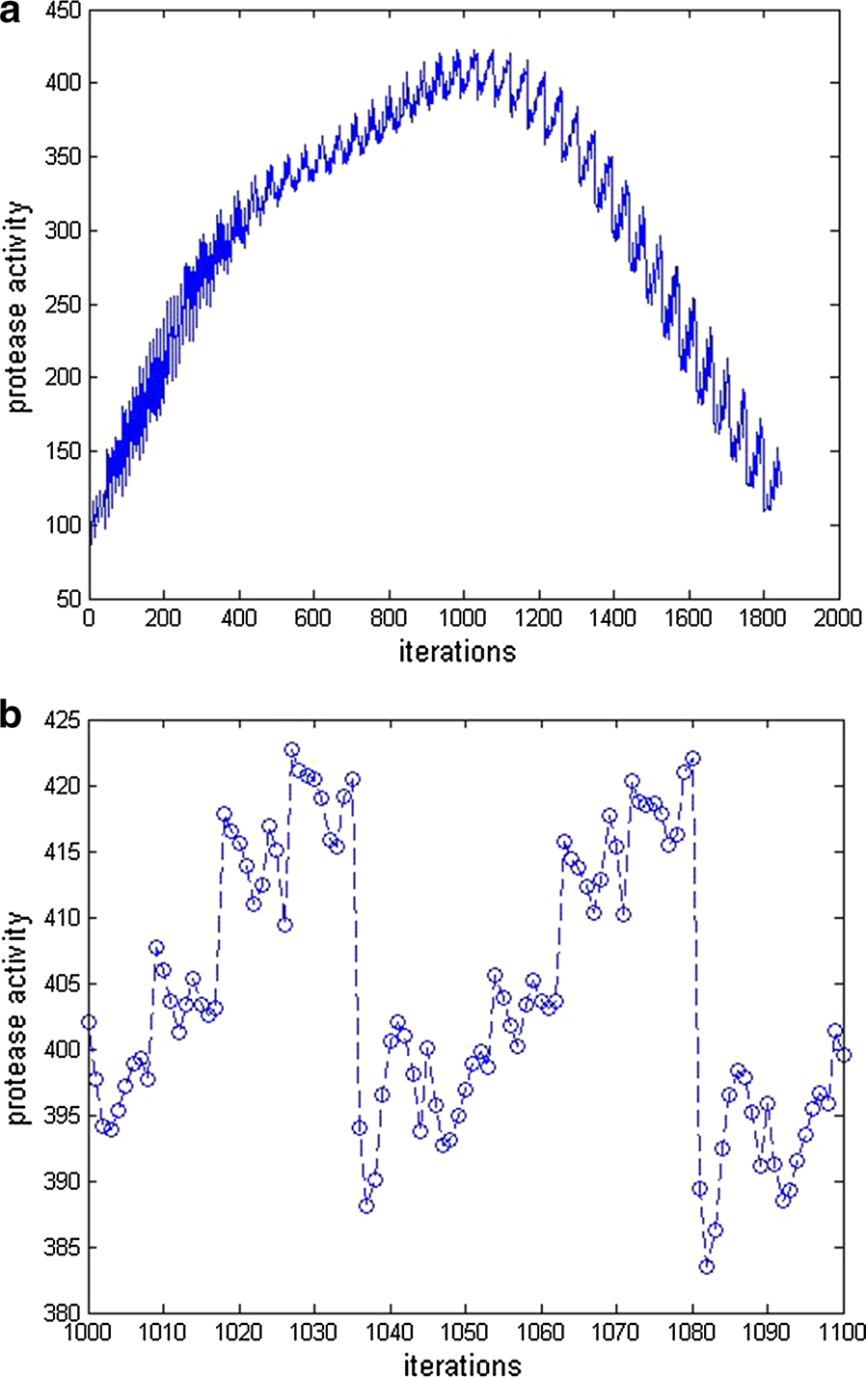
**a** Predicted protease activity response of the trained ANN network. **b** Region of maximum network response

Network output was calculated at 1850 possible combinations of inputs to achieve our goal of optimization of protease activity. The network output has displayed in Fig. 2a, b. Individual combination of temperature, pH and WB% was given an iteration number which was plotted in x-axis and the corresponding protease activity of network output was plotted in y-axis as shown in the Fig. 2a.

The maximum protease activity was between 1000th and 1100th iterations. The network response of between these values was further plotted in the Fig. 2b. It was found again that maximum protease activity was in between 1020th and 1030th iteration. The network response for these values is shown in the Table 3.

**Table 3:** Network response of maximum protease activity between 1020th to 1030th iteration

Iterations	1021	1022	1023	1024	1025	1026	1027	1028	1029	1030
Temperature	29	29	29	29	29	29	30	30	30	30
percentage of WB	77.5	80	82.5	85	87.5	90	70	72.5	75	77.5
pH	6.2	6.2	6.2	6.2	6.2	6.2	6.2	6.2	6.2	6.2
Protease activity, U/gds	413.82	411.02	412.42	416.95	415.1	409.35	422.66	421.14	420.72	420.41

## Results

The present study showed that out of the five substrates-wheat bran, rice husk, soybean meal, black gram husk and mixture of wheat bran and soybean meal; maximum alkaline protease production was observed by using mixture of wheat bran and soybean meal. The optimum ratio of wheat bran and soybean meal was found to be 4:1 (in g). The optimization of physiological parameters was initially done with one ‘factor at time method’. First incubation period was varied keeping other factors constant and maximum protease activity was observed after 72 h of incubation. A rapid reduction in protease activity was observed when the incubation temperature was raised beyond 28 °C. *Rhizopus oryzae* SN-5 strain secreted maximum amount of alkaline proteases at pH = 6. The nitrogen requirement of the strain for maximum secretion of proteases was found to be satiated by 1% sodium nitrate.

Based on the above findings, ratio of wheat bran to soybean meal, pH and temperature were selected as the critical process parameters and their optimum values were taken as initial search level in EVOP factorial design technique. Following the procedure, as described earlier, the design of three variables system has given in Table 1. The results of phase I of investigation have been shown in Table 4 where A1 and A6 represent the initial optimum conditions for the three parameters for the maximum production of alkaline protease. For the new sets of experiments, each variable parameter possessed two levels of magnitude, one lower and one higher, which were as follows: temperature (28 ± 2)  °C, pH (6 ± 2) and ratio of wheat bran (WB) to soybean meal (SM) (4:1 ± 0.5). As shown in Table 5, the calculations of the effects and analysis of the results of cycle I and cycle II for protease production showed that the changes in the main effect were larger and negative (− 231.94, − 214.54) and all the effects were smaller than the error limits (± 101.04, ± 93.40). The maximum production of protease was obtained at initial optimum conditions i.e. A6. From this analysis, it was concluded that optimum conditions for the production of alkaline proteases have been achieved. The yield of alkaline protease was 412.79 U/gds under the optimized conditions.

**Table 4:** Experimental conditions and results of experimental setup I for protease production

Parameters	A1	A2	A3	A4	A5	A6	A7	A8	A9	A10
Temperature/°C	28	26	26	30	30	28	30	26	30	26
pH	6	4	8	4	8	6	8	4	4	8
WB +SM ratio (in g)	4:1	3.5:1.5	4.5:0.5	4.5:0.5	3.5:1.5	4:1	4.5:0.5	4.5:0.5	3.5:1.5	3.5:1.5
Protease activity (U/gds) (Cycle 1)	406.17	158.97	48.63	88.80	153.34	412.79	189.32	107.69	110.26	99.30
Protease Activity (U/gds) (Cycle 2)	389.89	129.93	50.12	98.69	134.86	384.16	200.94	122.02	103.69	110.53
Differences	16.28	29.04	− 1.49	− 9.89	18.48	28.63	− 11.62	− 14.33	6.57	− 11.23
Average	398.03	144.45	49.38	93.74	144.10	398.47	195.13	114.85	106.97	104.91

**Table 5:** Calculations of effects and error limits for cycles I and II for amylase and protease

	Protease (Cycle 1)	Protease (Cycle 2)
Effects		
Effect of temperature (T)	− 26.05	− 7.55
Effect of pH (P)	11.70	7.11
Effect of WB +SM ratio (R)	30.50	40.56
Effect of TP	13.22	13.81
Effect of TR	34.59	28.93
Effect of PR	61.39	50.43
Change in mean effect	− 231.94	− 214.54
Standard deviation	100.65	93.03
Error limits		
For average	± 142.32	± 131.54
For effects	± 101.04	± 93.40
For change in mean	± 89.68	± 82.89

Further data obtained in EVOP has been considered as input data in ANN and used for training of software also. On processing the data in ANN, optimum conditions of pH, temperature and substrate that accounts for maximum protease activity of 422.66 U/ml were: temperature- 30 °C, optimum WB substrate percentage in a mixture of WB- 70% and optimum pH-6.2. The Levenberg–Marquardt algorithm solves the problems existing in both gradient descent method and the Gauss–Newton method for neural-networks training, by the combination of these two algorithms. The protease activity was found increased about 1.5 times from the experimental results. The validation result was also found fit to the result of ANN. The result of ANN and EVOP matches statistically. The results of EVOP technique are as shown in Tables 4 and 5.

Yield of alkaline protease was 412.79 U/gds under the optimized conditions obtained through EVOP technique and 422.66 U/ml with ANN optimization methodology, which is higher than EVOP.

## Discussion

The aim of present study was to optimize physiological conditions for maximum production of alkaline proteases from cost-effective and readily available substrates. Wheat bran and soybean meal both are agro industrial wastes that are low-cost and highly ubiquitous. During optimization studies, it was observed that fermentation carried out using a mixture of wheat bran and soybean meal produced maximum protease as compared to when wheat bran and soybean meal were used alone. The texture, porosity and carbohydrate content of wheat bran supports the growth of the organism, however, addition of protein rich soybean meal enhanced the protease production by 17%. While optimizing the ratio of wheat bran to soybean meal to be used in fermentation, it was observed that protease production decreased with the increase in the fraction of soybean meal. This observation may be explained by the presence of a number of protease inhibitors in soybean. Wheat bran and soybean meal have been used as substrates individually by various researchers for production of proteases (Chandran et al. 2005; Qazi et al. 2008). Omrane et al. 2018 obtained good protease activity from *Penicillium* sp. using wheat bran supplemented with 25 mg of soy protein per gram wheat bran. In another study conducted by Qazi et al. 2008, a mixture of wheat bran and soybean in ratio 8:2 (in g) was used for enhanced production of proteases using *Aspergillus niger*. Alkaline protease production by *Rhizopus* sp. utilizing a mixture of wheat bran and soybean has not been explored earlier.

Protease activity was increased drastically by inducing the organism prior to fermentation. This is done merely to reduce the lag phase of the organism. It was found that induction of the strain by using 1% casein and 0.5% glucose increased the protease activity by 2.14-fold. This is the most economical and effortless method for induction of the strain to improve the yield of enzyme, without deteriorating the quality of the product as well as that of the organism.

Alkaline protease activity was highest after 72 h of incubation. A rapid decline in protease activity was observed after 96 h. Protease degradation after maximum enzyme production. The accurate mechanism underlying this type of reduction in protease synthesis is not properly understood. However, autoproteolysis and high vulnerability of proteases to various inhibitors are hypothesized to be the major reasons behind the reduction of protease activity.

Temperature and pH play a pivotal role in production of extracellular enzymes. Alkaline protease production was found to be highest at 30 °C for the growth of this strain. At pH = 6.2, the secretion of protease was maximum, this suggests that the organism is able to produce proteases in slightly acidic conditions. These physiological conditions are ideal for the utility of this strain in protease production for applicability in detergent, leather and food-processing industries.

In order to achieve optimum physicochemical conditions for maximum yield of protease, three approaches: one factor at time, EVOP and ANN were assessed in current study. Yield of protease was highest 422.6 U/gds at physicochemical conditions obtained through ANN, because ANN method enables determination of interaction effects and error limits by making a small variation around the initial optimum conditions, which is not possible in other methods. ANN is simple, time-effective and highly reliable method as it does not require alteration in the existing plant and also poses no risk to product’s quality. Negi and Banerjee (2006) optimized parameters such as temperature, pH and relative humidity for the concomitant production of protease and amylase from *Aspergillus awamori* through EVOP methodology.

A number of reports are available on alkaline proteases production from microbial sources. But, micro-organisms with superior growth kinetics and inexpensive medium requirements producing highly stable proteases in short amount of time (within 72 h) are not well-known using agro industrial wastes such as wheat bran and soybean meal. In the current comparative study, ANN methodology was found better and cheaper way of optimization methodology than EVOP method. Yield of protease was found maximum at optimum physicochemical conditions obtained through ANN.

This work has demonstrated the use of ANN to optimize the culture condition of *Rhizopus oryzae* for the optimum yield of alkaline protease. The experimental design of ANN maximizes the amount of information that can be obtained, while reducing the number of individual experiments needed to be performed. It is less-time consuming and can be utilized for optimization of various fermentation processes. This study confirms the immense potential of *Rhizopus oryzae* SN-5 strain to produce large amount of alkaline proteases at low cost in comparatively shorter time.

## Data Availability

It is the responsibility of each author for providing authenticated data and material and data would be made available also.

## References

[CR1] Benabda O, Mhir S, Kasmi M, Mnif W, Hamdi M (2019). Optimization of protease and amylase production by *Rhizopus oryzae* cultivated on bread waste using solid-state fermentation. J Chem.

[CR2] Chandran S, Alagarsamy S, Szakacs G, Pandey A (2005). Comparative evaluation of neutral protease production by *Aspergillus oryzae* in submerged and solid-state fermentation. Process Biochem.

[CR3] Craik CS, Page MJ, Madison EL (2011). Proteases as therapeutics. Biochem J..

[CR4] Djamel C, Ali T, Nelly C (2009). Acid protease production by isolated species of *Penicillium*. Eur J Sci Res.

[CR5] Donnelly S, Dalton JP, Robinson MW, Robinson MW, Dalton JP (2011). How pathogen-derived cysteine proteases modulate host immune responses. Cysteine proteases of pathogenic organisms. Advances in experimental medicine and biology.

[CR8] Lowry OH, Rosenbrough NJ, Farr AL, Randall RJ (1951). Protein estimation with the Folin-phenol reagent. J Biol Chem.

[CR9] Mamo J, Assefa F (2018). The role of microbial aspartic protease enzyme in food and beverage industries. J Food Qual.

[CR10] Mazaa PK, OliveiraP Toledo MS, Paula DMB, Takahashi HK, Straus AH, Suzukia E (2012). Paracoccidioides brasiliensis induces secretion of IL-6 and IL-8 by lung epithelial cells modulation of host cytokine levels by fungal proteases. Microbes Infect.

[CR11] Milala MA, Jatau IA, Abdulrahman AA (2016). Production and optimization of protease from *Aspergillus niger* and *Bacillus subtilis* using response surface methodology. J Biotechnol Biochem.

[CR12] Neethu SK, Sreeja Devi PS, Arun SN (2016). A review on microbial proteases. Int J Adv Res.

[CR13] Negi S, Banerjee R (2006). Optimization of amylase and protease production from *Aspergillus awamori* in single bioreactor through EVOP factorial design technique. Food Technol Biotechnol.

[CR14] Omrane BM, Moujehed E, Ben EM, Mechri S, Bejar S, Zouari R, Baffoun A, Jaouadi B (2018). Production, purification, and biochemical characterization of serine alkaline protease from *Penicillium chrysogenum* strain X5 used as excellent bio-additive for textile processing. Int J Biol Macromol.

[CR15] Pandey AK, Edgard G, Negi S (2016). Optimization of concomitant production of cellulase and xylanase from *Rhizopus oryzae* SN5 through EVOP-factorial design technique and application in Sorghum Stover based bioethanol production. Renew Energy.

[CR16] Patil M, Shastri NV (1981). Extracellular proteases by Alternasia alternater (Fr.). J Ferment Technol..

[CR17] Potempa J, Pike RN (2009). Corruption of innate immunity by bacterial proteases. J Innate Immun..

[CR18] Protein Hydrolysis Enzymes Market by Sources (Microorganisms, Animals, Plants), Applications (Detergent Industry, Pharmaceuticals, Food Industry and Others), & Geography (North America, Europe, Asia-Pacific & ROW)—Global Trends & Forecasts to 2019

[CR19] Qazi JI, Jamshaid H, Nadeem M, Ali SS (2008). Production of proteases by *Aspergillus niger* through solid state fermentation. Punjab Univ J Zool..

[CR20] Román RC, Hernández OG, Urtubia UA (2011). Prediction of problematic wine fermentations using artificial neural networks. Biopro Biosyst Eng.

[CR21] Sattar AQ, Aqeel MB, Imrana K, Umar MD (2011). Optimization of cultural conditions for protease production by *Bacillus subtilis* EFRL 01. Afr J Biotechnol.

[CR22] Sharma M, Gat Y, Arya S, Kumar V, Panghal A, Kumar A (2019). A review on microbial alkaline protease: an essential tool for various industrial approaches. Ind Biotechnol.

[CR23] Vats S, Negi S (2013). Use of artificial neural network (ANN) for the development of bioprocess using *Pinus roxburghii* fallen foliages for the release of polyphenols and reducing sugars. Bioresour Technol.

[CR24] Walter HE, Bergmeyer HU, Bermeyer J (1984). Proteinases: methods with hemoglobin, casein and azocoll as substrates. Methods of enzymatic analysis.

[CR25] Yadav M, Sehrawat N, Sangwan A, Kumar S, Beniwal V, Singh AK (2013). Artificial neural network (ANN): application in media optimization for industrial microbiology and comparison with response surface methodology (RSM). Adv Appl Sci Res..

